# P02 ThinkJIA: A campaign to raise awareness that children and young people get arthritis, enabling families and frontline health professionals to recognise symptoms and refer to specialist services

**DOI:** 10.1093/rap/rkac067.002

**Published:** 2022-09-28

**Authors:** Richard Beesley

**Affiliations:** Juvenile Arthritis Research, Tonbridge, United Kingdom; European Network for Children with Arthritis, Geneva, Switzerland

## Abstract

**Introduction/Background:**

Awareness that Children and Young People (CYP) may develop arthritis (Juvenile Idiopathic Arthritis, JIA) is low within the general population and amongst non-rheumatology frontline health professionals. This can lead to delays in diagnosis and reduced access to treatment, with consequential risk of joint damage and permanent eye complications through undiagnosed JIA-associated uveitis. The aim of this work was to develop a campaign to enable frontline health professionals and families to be aware that CYP can develop arthritis, to know the main signs and symptoms, and to pursue early referral to speciality services.

**Description/Method:**

An awareness-raising campaign was developed by UK charity Juvenile Arthritis Research working with parents of CYP with JIA, adults with JIA, teachers, campaigners, paediatric rheumatologists, ophthalmologists, and other interested lay and professional individuals. The campaign, called #ThinkJIA, includes postcards with key messages and a supporting website, www.thinkjia.org

Resources are aimed at both the general population, to help improve recognition of the signs and symptoms and encourage early attendance at primary care health services; and at non-rheumatology frontline health professionals to support recognition of JIA symptoms and the need for prompt referral. Whilst the key messaging is common to both audiences, the language used differs to reflect the lay and medical professional expectations.

Once draft campaign resources were developed, they were shared with clinical professionals and tested by parents and school teachers.

**Discussion/Results:**

Of the pilot group of parents and teachers receiving draft resources, 100% of parents and 68% of teachers agreed that they would feel confident in seeking medical attention if their child, or a child in their school, started showing signs of JIA. Following feedback from recipients in the pilot group resources have been updated with clearer imagery. These are also supported by posters for use in health clinics and general circulation.

**Key learning points/Conclusion:**

The development of the #ThinkJIA awareness-raising resources has helped ensure frontline health professionals and the general population can have access to information about JIA. This can help facilitate early referral and improved access to treatment. The broad rollout of awareness resources is essential to appropriately support CYP with JIA and ensure they receive the treatment they require promptly.

**Acknowledgements:**

Thanks to Prof Helen Foster for her vital role in the initial development of these resources, and to all those involved in the development and pilot testing.

